# Resolution of Tonsillar Herniation and Syringomyelia Following Resection of a Large Anterior Frontal Parasagittal Meningioma

**DOI:** 10.7759/cureus.7636

**Published:** 2020-04-11

**Authors:** Sabastian Hajtovic, Dimitris G Placantonakis

**Affiliations:** 1 Neurosurgery, City University of New York (CUNY) School of Medicine, Sophie Davis Biomedical Education Program, New York, USA; 2 Neurosurgery, New York University (NYU) School of Medicine, New York, USA

**Keywords:** tonsillar herniation, syringomyelia, meningioma, chiari-i malformation

## Abstract

Chiari I malformation is the herniation of cerebellar tonsils below the level of the foramen magnum due to congenital or acquired pathologies. Acquired Chiari I malformation (ACM) may occur secondary to space-occupying lesions (SOLs), such as intracranial tumors due to elevated intracranial pressure (ICP), and can be accompanied by syringomyelia. ACM and syringomyelia have been shown to resolve after resection of the SOL, without the need for adjuvant posterior fossa decompression. The vast majority of SOLs leading to ACM have been reported in the posterior fossa, thus exerting a direct mass effect on the cerebellum. Supratentorial SOLs leading to ACM are much less frequent but, when present, are most commonly parieto-occipital. We report a rare case of a large anterior left frontal, parasagittal meningioma causing ACM and syringomyelia. These findings resolved following the resection of the meningioma, with no further surgical intervention. Our case demonstrates that ACM can occur secondary to an anterior supratentorial mass and further supports the idea that decompression of the posterior fossa is not required for the resolution of intracranial tumor-associated ACM and syringomyelia.

## Introduction

Chiari I malformation (CIM) refers to the caudal herniation of the cerebellar tonsils for a variable distance below the foramen magnum. This displacement may restrict the flow of cerebrospinal fluid (CSF), resulting in syringomyelia [1]. CIM may be associated with an underdeveloped posterior fossa as well as arachnoid pathologies of the foramen magnum and fourth ventricle [2]. Surgery to decompress the posterior fossa is often indicated if the CIM causes symptoms of the spinal cord or medullary compression, significant pain secondary to increases in intracranial pressure (ICP), or neurologic deficits linked to syringomyelia [1].

Acquired Chiari I malformation (ACM) may arise secondary to space-occupying lesions (SOLs) such as meningiomas. In these patients, the SOL may be resected, resolving the ACM and syringomyelia without the need for posterior fossa decompression. In the majority of reported cases, SOLs are located in the posterior fossa or parieto-occipital region [3]. Here, we present an unusual case of ACM and syringomyelia that completely resolved following the resection of a large frontal parasagittal meningioma.

## Case presentation

The patient is a 39-year-old, right-handed female with no significant medical history, who presented with progressive bi-frontal headaches for four months. She stated that her headaches were worse in the evening and frequently woke her up at night. She also complained of blurry vision. There were no focal findings on neurological examination.

**Figure 1:**
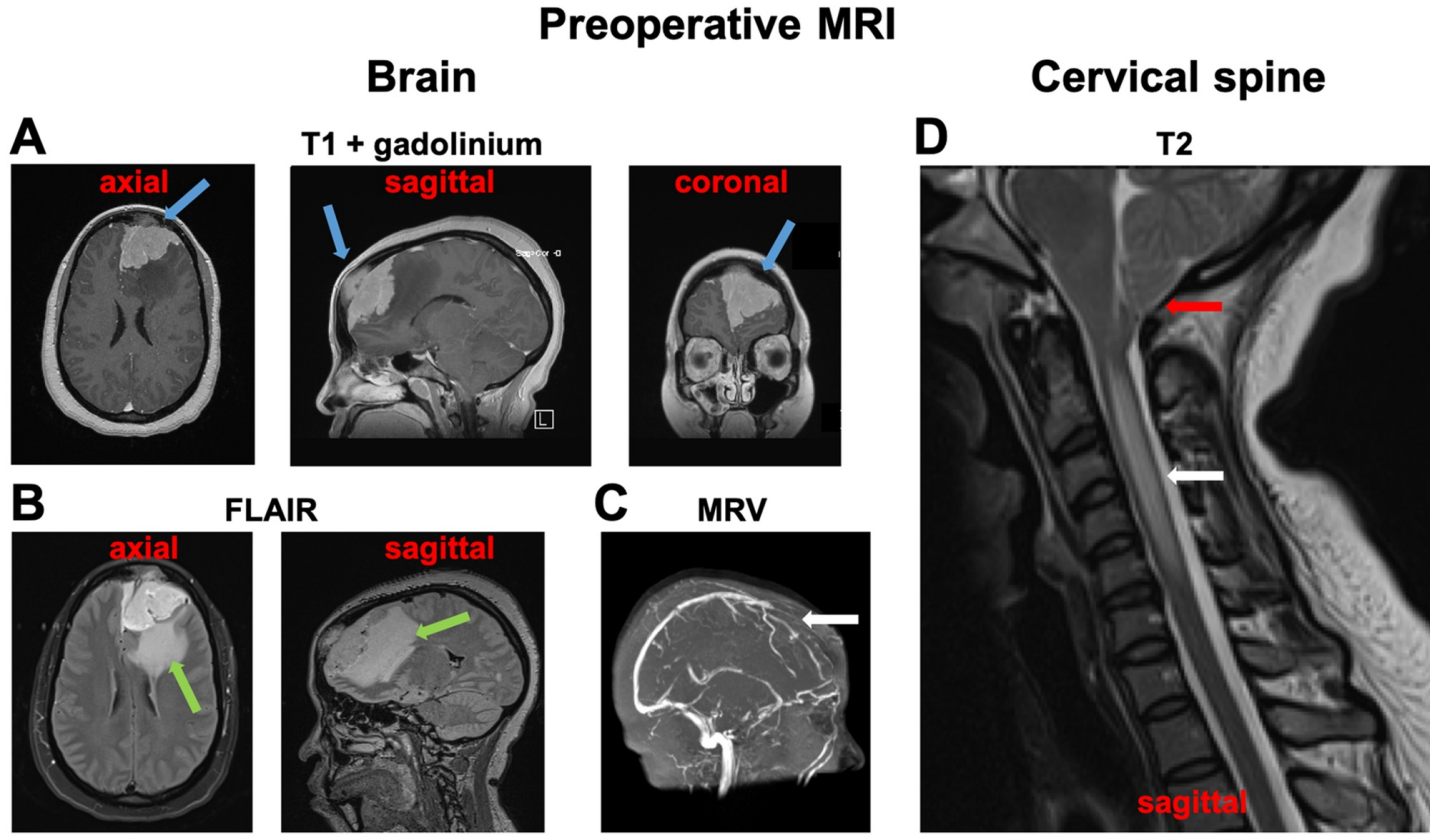
Preoperative MRI imaging A. Post-gadolinium T1-weighted images of the tumor (blue arrows). Note the calvarial involvement. B. Fluid-attenuated inversion recovery (FLAIR) imaging shows extensive peritumoral edema (green arrows). C. MR venogram (MRV) showed the occlusion of the superior sagittal sinus at the level of the tumor (white arrow). D. Sagittal T2 imaging of the cervical spine demonstrates tonsillar herniation (red arrow) below McRae’s line and cervical syringomyelia (white arrow).

Her symptoms prompted an ophthalmologic examination, which was notable for papilledema. Magnetic resonance imaging (MRI) showed a large, left, medial-frontal enhancing extra-axial tumor, consistent with meningioma, which measured 7.1 cm in its longest diameter and invaded the frontal calvaria (Figure 1A). There was significant vasogenic edema (Figure 1B). The tumor also invaded and occluded the superior sagittal sinus (Figure 1C). In addition, the MRI demonstrated cerebellar tonsillar herniation and cervical syringomyelia (Figure 1D). The caudal descent of the cerebellar tonsils was 15.9 mm below the level of the foramen magnum (McRae’s line). The rostrocaudal extent of the cervical syrinx was 56.7 mm.

**Figure 2:**
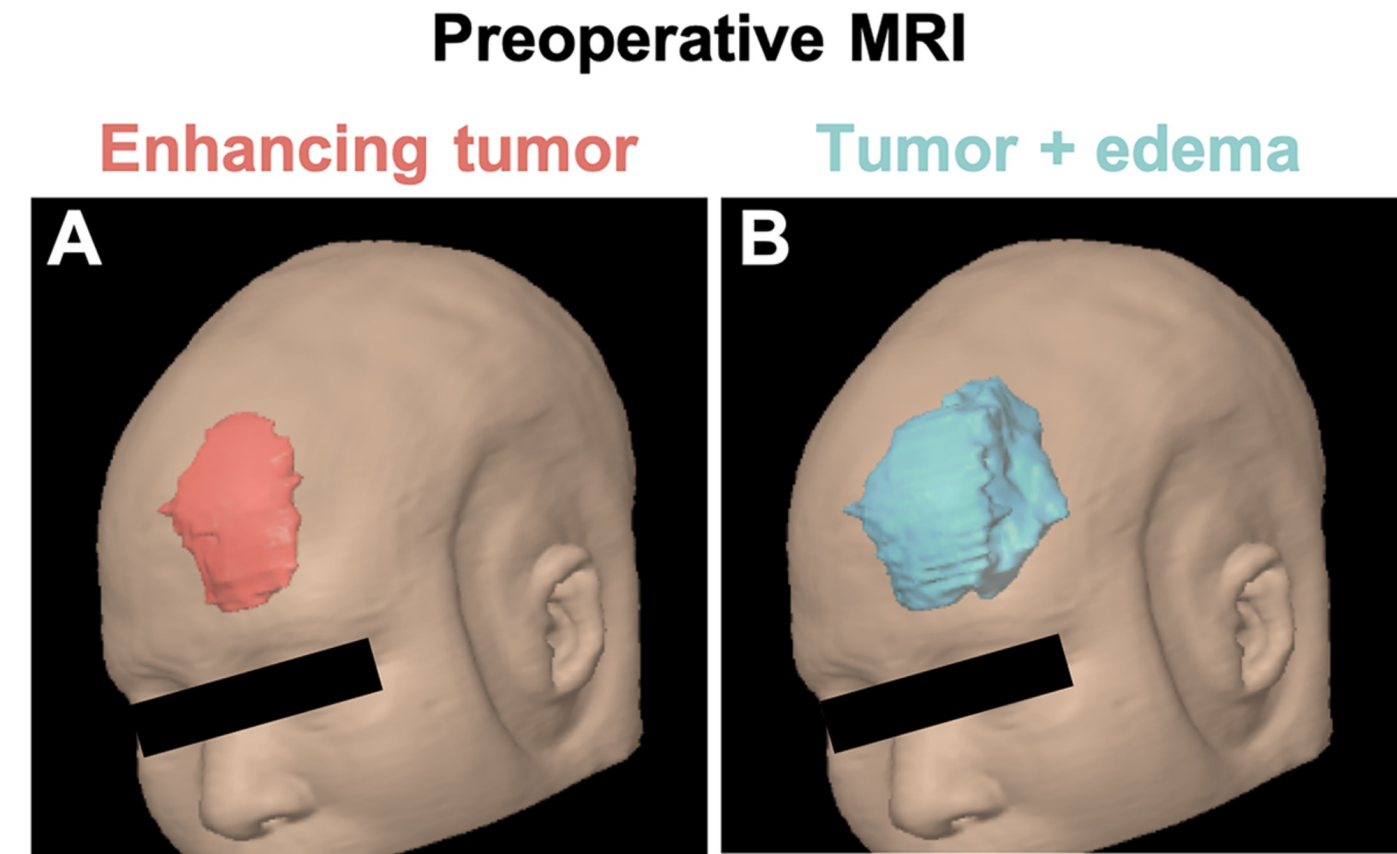
Three-dimensional models of the tumor and peritumoral edema Volumetric approaches on the BrainLab platform allowed us to quantify the volume of the tumor alone (A) or combined with the surrounding edema (B), as well as to visualize them on three-dimensional models. BrainLAB AG: Munich, Germany

Using volumetric approaches and BrainLab software (BrainLAB AG, Munich, Germany), we measured the size of the tumor, excluding the intraosseous component, as 44.715 cm^3^ (Figure 2A). When accounting for vasogenic edema in addition to the tumor, the volume increased to 106.027 cm^3^ (Figure 2B).

**Figure 3:**
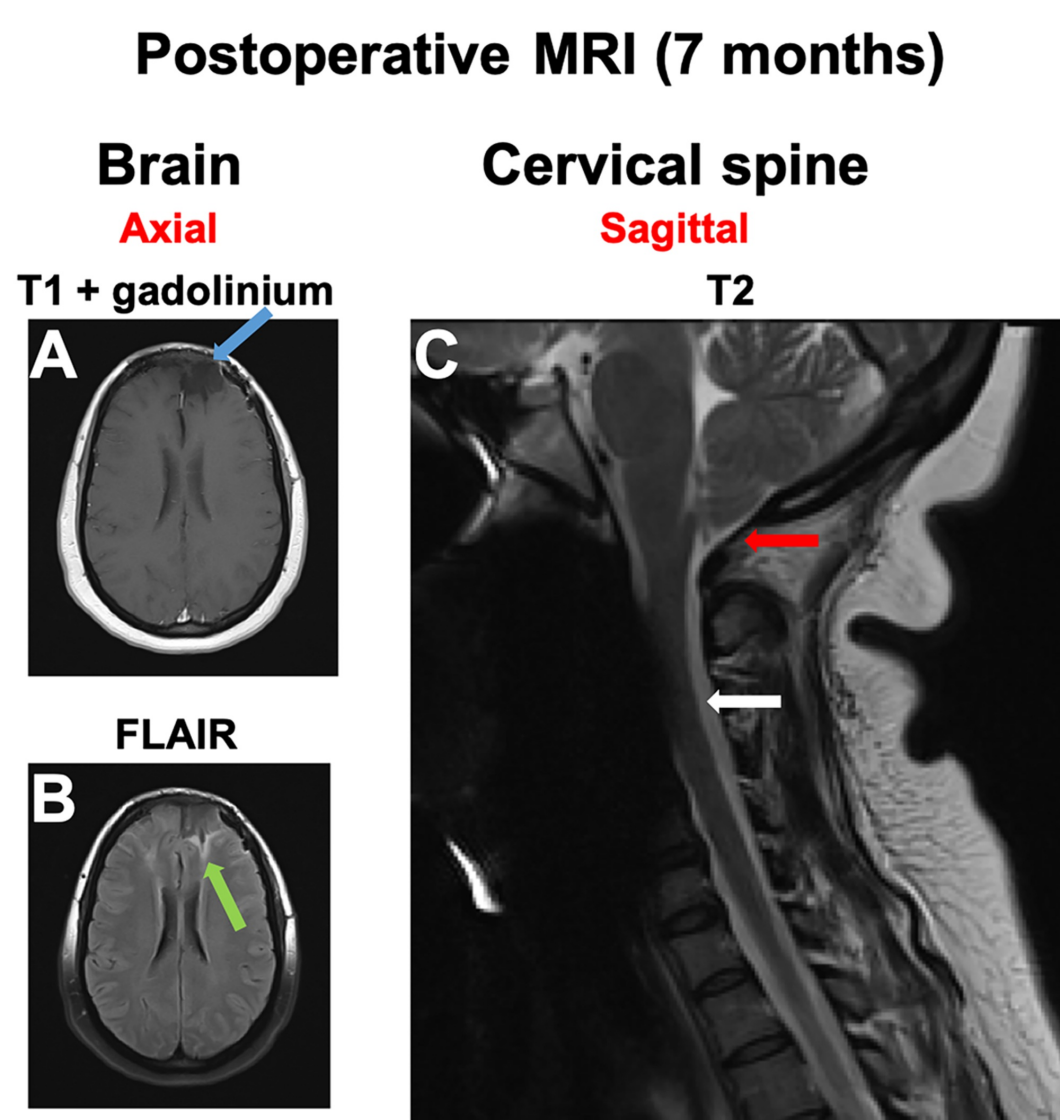
Postoperative MRI imaging A,B. At seven months after the procedure, MRI of the brain (left) showed (A) no tumor recurrence (blue arrow on T1 + gadolinium image) and (B) near-complete resolution of the peritumoral edema (green arrow on FLAIR image). C. The cervical spine MRI demonstrated resolution of the tonsillar herniation (red arrow) and cervical syrinx (white arrow). FLAIR: fluid-attenuated inversion recovery

Our patient underwent gross total resection of the meningioma with excision of the involved superior sagittal sinus. We also performed titanium mesh cranioplasty to repair the craniectomized calvaria that was involved with the tumor. Pathology was consistent with World Health Organization (WHO) grade II atypical meningioma. Postoperative MRI seven months later demonstrated near-complete resolution of the peritumoral edema around the resection cavity (Figures 3A-3B), as well as the resolution of the ACM and syringomyelia (Figure 3C). A follow-up examination demonstrated the absence of all preoperative neurologic symptoms.

## Discussion

CIM is classically thought to be a congenital anomaly in which hydrocephalus was originally implicated in the tonsillar herniation [4]. Other studies have linked CIM to a mismatch in the volume of the cerebellum relative to the posterior fossa, although normal fossa dimensions can also be seen [4]. Tonsillar herniation has also been shown to occur following CSF removal by lumbar shunting or multiple lumbar punctures [5]. This is likely due to the resulting craniospinal pressure gradient, which is directed toward the lower intraspinal pressure, causing caudal herniation of the cerebellar tonsils [4,6]. In addition, syringomyelia formation may be driven by functional obstruction at the foramen magnum, leading to increased intracranial and cervical CSF pulse pressure relative to the subarachnoid space [3,7]. However, the exact mechanism of syringomyelia formation remains unclear, as several pathogenic processes have been proposed, ranging from altered CSF flow dynamics to reduced cervical venous compliance, leading to the accumulation of extracellular fluid in the cervical spinal cord [8].

Wang et al. conducted a systematic review of surgical management of ACM secondary to SOLs [3]. Specifically, the authors examined the differences in clinical outcomes for patients who underwent SOL resection with or without posterior fossa decompression. Decompression is most commonly done with suboccipital craniectomy and C1 laminectomy [1,9]. In nearly all cases in both surgical groups, as seen in our patient, syringomyelia resolved or improved with resection of the SOL without requiring a syrinx shunt [3]. Similarly, the authors did not find a meaningful difference in the radiologic resolution of tonsillar herniation between the two surgical groups.

In the same systematic review of 44 patients, 36% of lesions were meningiomas while 32% were arachnoid cysts [3]. Eighty-five percent of the SOLs were large and 89% were infratentorial [3]. The infratentorial location of such lesions is thought to cause ACM by a direct, compressive mass effect on the cerebellum in the posterior fossa [10]. In many cases, the presence of an SOL in the posterior fossa caused obstructive hydrocephalus [3]. Of the four supratentorial masses included in the review, one was a large, left posterior parasagittal meningioma, two were parieto-occipital meningiomas, and one was a cerebellopontine angle meningioma [11-14]. This is in contrast to our patient’s large parasagittal anterior frontal meningioma with the extensive peritumoral edema, which may not have been expected to cause an ACM due to its anterior position. However, as with the downward-directed craniospinal gradient seen in spinal shunts, the same relative gradient due to increased ICP from a large mass can promote tonsillar herniation [4,6]. Following resection of the SOL and resolution of the peritumoral edema, the dissolution of the craniospinal gradient allows for the ascent of the cerebellar tonsils. In turn, this ascent can decompress the subarachnoid space at the level of the foramen magnum, allowing for the resolution of the syringomyelia [6]. Importantly, in the case of tumor-associated ACM, decompression of the posterior fossa is typically not necessary for treatment. This was particularly true in the case presented here, where the ACM and syringomyelia were asymptomatic.

## Conclusions

ACM secondary to an intracranial tumor is a rare finding, with a lack of large prospective studies and only one systematic review on the topic. Furthermore, specific measurements of the size of SOLs causing ACM, as well as precise measurements of tonsillar descent, are not consistently reported in the literature. While the majority of reported cases involve infratentorial masses, our case demonstrates that a large anterior frontal tumor with significant vasogenic edema may increase the ICP enough to shift the craniospinal gradient and cause tonsillar herniation. Our case is also consistent with the notion that an adjuvant posterior fossa decompression surgery is not required for the resolution of ACM and syringomyelia secondary to tumors.
